# A novel promising diagnostic candidate selected by screening the transcriptome of Babesia gibsoni (Wuhan isolate) asexual stages in infected beagles

**DOI:** 10.1186/s13071-022-05468-4

**Published:** 2022-10-10

**Authors:** Jiaying Guo, Furong Yang, Lingna Wang, Xuenan Xuan, Junlong Zhao, Lan He

**Affiliations:** 1 State Key Laboratory of Agricultural Microbiology, College of Veterinary Medicine, Huazhong Agricultural University, Wuhan, 430070 Hubei China; 2 Key Laboratory of Preventive Veterinary Medicine in Hubei Province, Wuhan, 430070 Hubei China; 3 National Research Center for Protozoan Diseases, Obihiro University of Agriculture and Veterinary Medicine, Obihiro, Hokkaido 080-8555 Japan; 4 Present Address: Northeast Agricultural University, Harbin, 150000 Heilongjiang China

**Keywords:** *Babesia gibsoni*, Canine babesiosis, Transcriptome, BgP30, Diagnostic candidate

## Abstract

**Background:**

*Babesia gibsoni* is one of the causative agents of canine babesiosis worldwide. Some dogs infected with *B. gibsoni* show severe clinical signs with progressive anemia, hemoglobinuria and splenomegaly. However, most infected dogs present a state of chronic infection and thereby may be a persistent pathogen carrier, increasing the risk of pathogen spreading. To date, little is known about this pathogen, with genomic and transcriptomic data in particular generally unavailable. This lack of knowledge extensively limits the development of effective diagnostic strategies and vaccines.

**Methods:**

High-throughput RNA sequencing of total RNA of *B. gibsoni* asexual stages collected from infected beagles was performed. The unigenes were annotated in seven databases. The genes were sorted according to their fragments per kilobase per million (FPKM) value, which was used as an indicator for expression level. The gene with the highest FPKM value was cloned from the genome of *B. gibsoni* and further tested for immunogenicity, cellular localization and efficacy as a potential diagnostic candidate for detecting *B. gibsoni* in sera collected from beagles.

**Results:**

A total of 62,580,653 clean reads were screened from the 64,336,475 raw reads, and the corresponding 70,134 transcripts and 36,587 unigenes were obtained. The gene with the highest FPKM value was screened from the unigenes; its full length was 1276 bp, and it was named BgP30. The BgP30 gene comprised three exons and two introns, with a 786-bp open reading frame, and encoded 261 amino acids with a predicted molecular weight of 30 kDa. The cellular localization assay confirmed the existence of P30 protein in *B. gibsoni* parasites. Moreover, P30 was detected in the serum of experimentally *B. gibsoni*-infected beagles, from 15 days up to 422 days post-infection, suggesting its usefulness as a diagnostic candidate for both acute and chronic infections.

**Conclusions:**

We sequenced the transcriptome of *B. gibsoni* asexual stages for the first time. The BgP30 gene was highly expressed in the transcriptome screening experiments, with further studies demonstrating that it could induce immune response in *B. gibsoni*-infected dogs. These results lead us to suggest that bgP30 may be a good diagnostic candidate marker to detect both acute and chronic *B. gibsoni* infections.

**Graphical Abstract:**

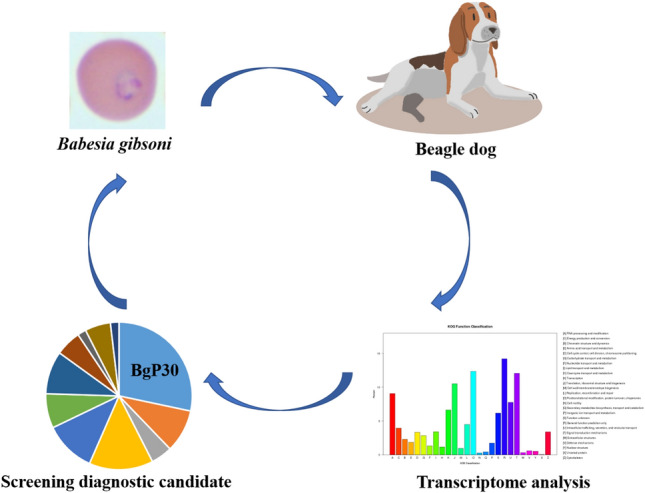

**Supplementary Information:**

The online version contains supplementary material available at 10.1186/s13071-022-05468-4.

## Background

Babesiosis as an emerging tick-borne zoonosis has received increasing attention due to its risks for severe clinical signs and even death. Canine babesiosis is an increasing babesiosis that occurs in dogs. Initially, the agents for canine babesiosis were reported according size, with the larger being *Babesia canis* and the smaller, *Babesia gibsoni* [1]. However, with increasing knowledge of these agents, some “*B. canis*” were found to be identical in size but different in vectors, clinical manifestations, pathogenicity and geographic distribution [2]. Improvements in molecular biology techniques has lead to the identification of subspecies of *B. canis*, including *B. canis*, *B. vogeli* and *B. rossi*, based on the 18S rRNA gene [3]. An additional two, yet unnamed “large” size species were also identified with different geographic distributions in eastern and southeastern USA in immunocompromised dogs and the UK, respectively [4–6]. Two new members have also been added one by one in to the “smaller” group, *B. conradae* and *B. microti*-like piroplasm (later named *B. vulpes*) [7–9]. Recently, a medium-sized (between the “large” and “smaller” size) *Babesia* species, named *B. negevi*, was identified [10]. Due to this great variety of *Babesia* spp., canine babesiosis is widely distributed all over the world with global significance. Of the known species, *B. gibsoni*, which was first recognized in India as a new piroplasm in 1910, is the causative agent for canine babesiosis and has been reported in several regions in Europe, Africa, Middle East, Americas, Australia and Asia [11]. In China, the first cases of canine babesiosis caused by *B. gibsoni* was documented by in 1985 [12]. Since then, canine babesiosis caused by *B. gibsoni* has been reported in succession in multiple cities and provinces in China, mainly in locations in the northern, eastern and central parts of China, including Liaoning Province in 2011, Jiangsu Province in 2014 and 2015, Shandong, Anhui, Zhejiang and Fujian provinces in 2015, Hubei Province in 2017, Jiangxi Province in 2015 and 2017, Henan Province in 2016 and 2019, Shaanxi Province in 2020 and the municipalities of Shanghai in 2015 and Beijing in 2017 [13–20].

*Babesia gibsoni* is a tick-borne pathogen and potentially transmitted by different tick species [21]. In addition to the traditional transmission route by ticks, however, an increasing number of clinical investigations indicate that this pathogen can be opportunistically transmitted by other routes, including, for example, blood transfusion, dog bites and transplacental routes [22–24]. Those multiple transmission routes may represent the main factor underlying the high the prevalence of *B. gibsoni*.

 The clinical manifestations for *B. gibsoni* infection are varied depending on the host’s age and immune status. Most dogs infected with *B. gibsoni* show mild signs of disease, whereas some will show severe signs with progressive anemia, hemoglobinuria, thrombocytopenia, splenomegaly and hepatomegaly [25]. In most cases, *B. gibsoni* infection is asymptomatic or at a stage of chronic infection after an acute infection, with low parasitemia persisting in host tissue for several years. In this latter case, *B. gibsoni* is rarely cleared from the host and, consequently, the host becomes a persistent carrier with the risk of pathogen spreading. As such, subclinical disease and low parasitemia pose a challenge for diagnosis. Currently, there are three known methods to diagnose *B. gibsoni* infection: direct microscopy study, immunological detection methods and molecular methods. The microscopy examination is the most common and fastest and is always used as the first way to detect *Babesia* spp. However, this pathogen prefers to hide in the peripheral circulatory system to evade the immune system of the host; thus, blood samples collected for microscopic detection may lack the parasite, leading to false-negative results [26, 27]. Immunology-based diagnosis is based on detecting antibodies that are produced in the infected dogs, and a number of *B. gibsoni* antigens have been identified to be relatively effective as diagnostic markers, such as BgSA1, BgP50 and thrombospondin-related adhesive protein (TRAP) [28–30]. However, antibodies can only be detected in cases of chronic infection as time is needed for the production of antibodies. Molecular methods consist mainly of the detection of *Babesia* DNA by PCR; as such these tests are more sensitive and faster than the other methods [26]. However, once again, it is hard to detect this parasite because it hides in the peripheral circulatory system; consequently, molecular methods have the same limitation as the microscopy examination. It is therefore necessary to combine different methods to detect the parasite. So far, there are a number of antigens of *B. gibsoni* that have been identified as potential targets for diagnosis, such as P32, P38, P50, P57 and P47 that have mostly been tested by enzyme-linked immunosorbent assay (ELISA) [31–34]. However, some of these were ineffective or showed lower sensitivity, resulting in almost no effective antigen that can be applied in clinical practice. Consequently, the diagnosis of *B. gibsoni* remains challenging.

The development of effective diagnostic candidates is closely related to a comprehensive understanding of *B. gibsoni* biology. The current lack of information on the genome and transcriptome of *B. gibsoni* extensively limits the elucidation of the *B. gibsoni* mechanisms of its life-cycle and thereby hinders the development of good diagnostic methods and vaccine candidates. In this article, we sequenced and analyzed the transcriptome of *B. gibsoni* (Wuhan isolate). We then selected an antigen that showed relative high expression to conduct subsequent assays to explore its potential to be an effective diagnostic candidate.

## Methods

### Experimental animals

Three 4-year-old healthy beagle dogs were purchased. They were subsequently tested and found to be free of *Babesia* infection using light microscopy, PCR (18S and internal transcribed spacer primers), real-time PCR and loop-mediated isothermal amplification (LAMP) methods [35, 36] (Additional file 1: Table S1).

Each beagle dog was injected intravenously with 1 ml of *B. gibsoni* (Wuhan strain)-infected blood that had been stored in liquid nitrogen (percentage parasitized erythrocytes [PPE]: 1%). At 1 week after injection, blood samples from each beagle were tested by PCR and examined by microscopy to monitor and calculate the PPE (Additional file 2: Figure S2). When PPE reached 15%, blood from the three beagle dogs was collected into sterile vacuum tubes containing anticoagulant with EDTA. After blood collection, each beagle dog was treated with diminazene aceturate and vitamins.

### Blood preparation

The procedure used for preparing blood in the present study was modified from a previous study [34]. The infected blood was centrifuged at 1500 rpm for 15 min at 4 °C, and the supernatant and layer of white blood cells removed completely. The pellet was resuspended in cold phosphate buffered saline (PBS) to the original volume and gently mixed. These steps were repeated three more times until the supernatant was clear and all white cells discarded [34].

The infected red blood cells (RBCs) were lysed by Red Blood Cell Lysis Buffer (Beyotime Biotechnology, Shanghai, China), following which the solution was left standing for 1 h at 4 °C. The RBCs were then completely lysed by strenuous vibration. The lysate was then centrifuged at 14,000 rpm for 10 min at 4 °C, and the supernatant was discarded. The pellet was washed in PBS, and the above steps were repeated until the color of the pellet was white rather than red or pink. The pellet was then resuspended in 1 ml PBS and filtered through a 2-μm nuclepore Track-Etch membrane filter (Whatman, Maidstone, UK). The effluent liquid was collected and kept for further *B. gibsoni* RNA extraction.

### RNA isolation, complementary DNA library construction and sequencing

The RNA of *B. gibsoni* from the blood collected from the three beagle dogs was individually isolated by TRIzol reagent (Life Technologies, Thermo Fisher Scientific, Waltham, MA, USA), extracted by chloroform, precipitated by isopropyl alcohol and ethanol and treated with DNase I (Invitrogen, Thermo Fisher Scientific), thus avoiding DNA contamination. The isolated RNA was processed with a preliminary quantitation, tested for degradation and potential contamination and checked for integrity and quantity. Total RNA was prepared by messenger RNA (mRNA) enrichment using oligo beads. The enriched mRNA was then fragmented randomly in fragmentation buffer, followed by complementary DNA (cDNA) synthesis using random hexamers and reverse transcriptase. Construction of the cDNA library needed for sequencing required a round of purification, terminal repair, tailing, ligation of sequencing adapters, size selection and PCR enrichment, as well as accurate quantification quantitative PCR. Sequencing of the cDNA library was performed on an Illumina Hiseq X Ten sequencing platform (Novogene Bioinformatics Technology Co., Beijing, China).

### Data analysis

The raw data obtained from the Illumina Hiseq X Ten sequencing platform were transformed to sequenced reads by base calling, followed by quality control, which included the evaluation of error rate, of GC content distribution and of data filtering. Due to the absence of the *B. gibsoni* genome as reference, clean reads were assembled to obtain a reference sequence for subsequent analysis using Trinity software [37]. The obtained sequences were processed with hierarchical clustering using the command-line program Corset. The RSEM software package was used to map reads back to the transcriptome and quantify the expression level, based on the FPKM (fragments per kilobase per million reads) value [38]. The annotation of the gene function was performed using several database: NR (NCBI non-redundant protein sequences), PDB (Protein Data Bank), Swiss-Prot, PIR (Protein Information Resource), PRF (Protein Research Foundation), COG (Cluster of Orthologous Groups of proteins), KOG (euKaryotic Orthologous Groups), GO (Gene Ontology) and KEGG (Kyoto Encyclopedia of Genes and Genome) [39–41]. CoDing Sequence (CDS) prediction was carried out by Basic Local Alignment Search Tool (BLAST; https:blast.ncbi.nlm.-nih.gov/Blast.cgi) unigenes based on the priority of the NR and Swiss-Prot databases. If there were no hits in BLAST, unigenes were analyzed using the ESTScan (3.0.3) program to predict their coding regions and determine their sequence direction. A single nucleotide polymorphism and insertion-deletion were detected using the Genome Analysis Toolkit (GATK3) [42], and simple sequence repeat detection of the unigenes was performed using the MISA (v1.0) tool. Quantitative real-time PCR was conducted to verify the results obtained by sequencing.

### Gene amplification and recombinant protein expression

Due to incomplete information on the genome of *B. gibsoni*, the sequence of the reference genome was also based on other *Babesia* species whose genome information had been submitted to the NCBI database, such as *Babesia bovis*, *Babesia ovata*, *Babesia bigemina*, *B. microti*, among others. The transcriptome level of the different genes was indicated by the FPKM value [43]. The genes were sorted by FPKM values; the one that was highly expressed at the transcriptome level was annotated to the hypothetical protein of *B. bovis* (NCBI reference sequence: XP_001611467) using the NR database. In order to obtain the gene sequence in *B. gibsoni*, the amino acid sequence of the hypothetical protein of *B. bovis* was regarded as the query sequence and BLAST was used to screen the high similarity sequence in the incomplete assembly genome of *B. gibsoni*. The gene thus obtained was analyzed using bioinformatic tools. Based on the obtained sequence in the genome, primers were designed to clone the gene in *B. gibsoni*. Due to the presence of two introns, the gene was truncated and expressed from 749 to 1276 bp. The truncated gene was amplified from the cDNA of *B. gibsoni* and then ligated with the pE-sumo vector to construct the recombinant plasmid by the homologous recombination method. The forward primer for cloning the *P30* gene was 5′-CAC CGC GAA CAG ATT GGA GGT CGG GAT CCA TAC GGC TAT GAG-3′ and the reverse primer was 5′-TCG AAT TCG GAT CCT CTA GTT CAG CGT GGC ACA CGA CGT TC-3′. The forward primer for cloning pE-sumo was 5′-ACC TCC AAT CTG TTC GCG GTG-3′and the reverse primer was 5′-ACT AGA GGA TCC GAA TTC GA-3′. The PCR cycling parameters for the *P30* gene were: an initial denaturation for 2 min at 98 °C; followed by 35 cycles of denaturation at 98 °C for 10 s, annealing at 55 °C for 10 s and extension at 72 °C for 10 s; with a final extension for 10 min at 72 °C. The PCR cycling parameters for the pE-sumo vector were: 95 °C for 3 min; followed by 35 cycles of 95 °C for 15 s, 56 °C for 15 s and 72 °C for 6 min; with a final extension for 1 min at 72 °C. The amplicon product for the *P30* gene was ligated to the pE-sumo vector by the ClonExpress II One Step Cloning Kit (Vazyme Biotech, Nanjing, China). The recombinant plasmid construct was confirmed by enzyme digestion and PCR for integration. The validated pE-sumo-P30 product was transfected into *Escherichia coli* BL21 (DE3) for recombinant protein expression. Based on the ratio of 1:100, the pE-sumo-P30 bacterial solution was transferred to LB medium containing Amp^+^ (1:1000) for incubation at 37 °C for about 3 h to reach an absorbance of 0.5 at A_600_. The pE-sumo-P30 was then induced by isopropyl-β-d-thiogalactopyranoside (IPTG) (Sigma-Aldrich, St. Louis, MO, USA) at 37 °C for 4 h and analyzed by sodium dodecyl sulphat-polyacrylamide gel electrophoresis (SDS-PAGE). The pE-sumo-P30 was His-tagged and purified by ProteinPure Ni–NTA Resin (TransGen Biotech, Beijing, China) and stored at − 80 °C.

### Polyclonal antibody production

Two Japanese white rabbits, aged 2–3 months, were prepared to generate antibodies against the recombinant BgP30 (rBgP30). For the first immunization procedure, 400 μg rBgP30 that had been completely mixed with an equal volume of Freund’s complete adjuvant (Sigma-Aldrich) was subcutaneously injected into the back of the rabbits. The rabbits were subsequently given three boosters (each 150 μg in Freund’s incomplete adjuvant) at 2-week intervals. The serum was collected before and 2 weeks after the final booster and the titers of antibody tested using ELISA.

### Enzyme-linked immunosorbent assay

Blood was collected from each beagle dog infected with *B. gibsoni* from day 1 up to day 422 post infection. The serum collected on the different days was collected separately and stored at − 20 °C. The concentration of rBgP30 was evaluated using the BCA Protein Assay Kit (Beyotime Biotechnology). rBgP30 was diluted by coating buffer (25 mM carbonate buffer solution, pH 9.6) to 1 μg/ml, and each well of the 96-well (flat-bottom) plate was coated with purified rBgP30 at 4 °C and allowed to stand overnight. The plate was then blocked by 1% bovine serum albumin (BSA) at 37 °C for 30 min, followed by incubation with the serum collected on different days post infection, diluted 1:1000 in 0.1% BSA/PBS, with each well containing 200 μl, at 37 °C for 1 h. The secondary antibody, horseradish peroxidase (HRP)-labeled goat anti-canine (Beyotime Biotechnology), diluted 1:5000 in 0.1% BSA/PBS, was added to each well at 37 °C for 1 h. Then, TMB (Sigma-Aldrich), a HRP substrate, and H_2_O_2_ were added to each well, and the reaction was stopped by the addition of 0.25% hydrofluoric acid. The absorbance values were measured at OD630.

### Immunodetection of rBgP30

The rBgP30 protein was separated by SDS-PAGE and transferred to a PVDF membrane (Thermo Fisher Scientific), then blocked with 5% skim milk at 4 °C overnight. The membrane was subsequently incubated with the serum of *B. gibsoni*-infected beagle dogs diluted 1:500 in PBS at 37 °C for 3 h. The secondary antibody, HRP-labeled goat anti-canine (Beyotime Biotechnology), was incubated with the membranes. The membranes were visualized using diaminobenzidine (ZSGB-BIO, Beijing, China).

### Immunofluorescence antibody assay

Polyclonal antibody was prepared for detection of BgP30 in the *B. gibsoni* by immunofluorescence antibody assay (IFAT). The RBCs from *B. gibsoni*-infected beagle dogs was smeared onto the slide. The smears were fixed with 95% methanol and 5% acetone (v:v) at − 20 °C for 30 min, permeabilized for 10 min with Triton X-100 in PBS and then washed three times in PBS. The rabbit polyclonal antibodies against rP30 diluted 1:500 in 1% BSA/PBS were added to the smears at 37 °C for 2 h. The control groups were incubated with the serum of naïve rabbits. After the smears were washed three times in PBS, they were simultaneously incubated with the Alexa Fluor 488-conjugated secondary antibody (Life Technologies, Thermo Fisher Scientific) diluted 1:2000 in 1% BSA/PBS as the secondary antibody and Hoechst (Invitrogen, Thermo Fisher Scientific) for nuclear staining at 37 °C for 1 h in the dark. The slides were analyzed by fluorescence microscopy or stored at 4 °C for further analysis.

## Results

### RNA sequencing and de novo assembly

**Fig. 1:**
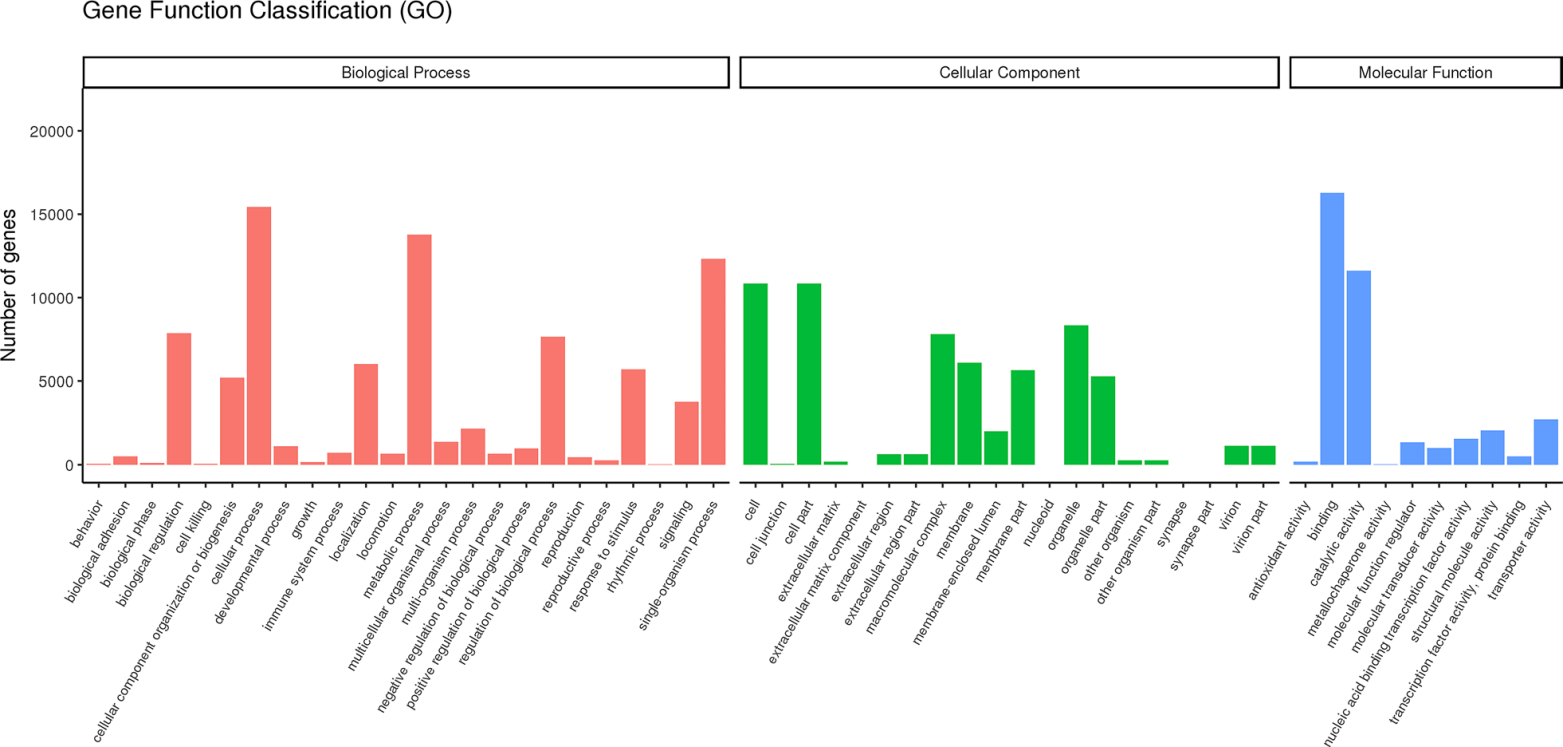
Gene function classification of transcriptome data on *Babesia gibsoni* during the asexual stages in infected beagle dog. Red indicates the biological process category, green indicates the cellular component category and blue indicates the molecular function category

**Fig. 2:**
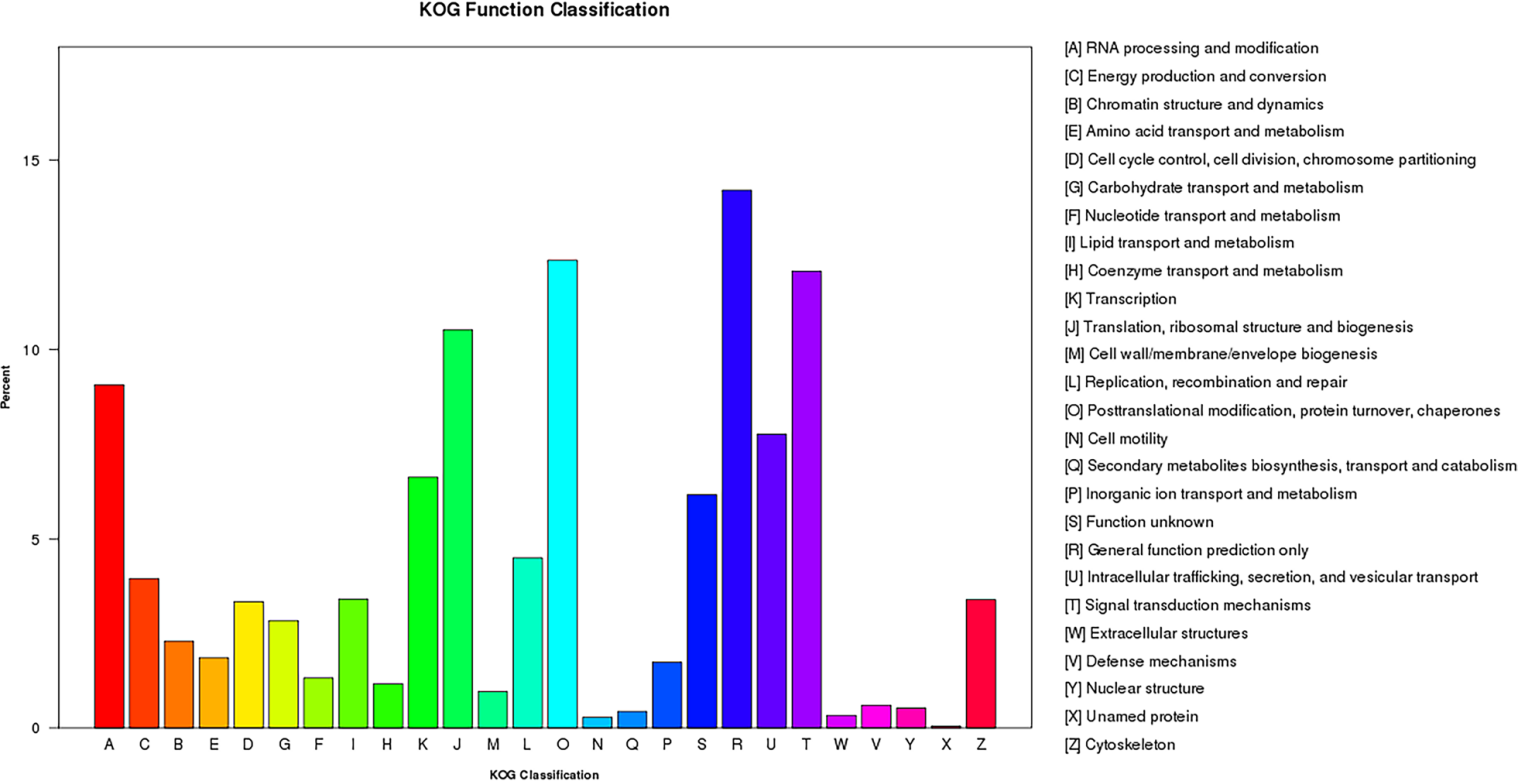
KOG function classification. A total of 26 groups of KOG functions were identified in the transcriptome data of *B. gibsoni*. The percentage of genes that annotated to the groups are shown on the* Y*-axis. KOG, euKaryotic Orthologous Groups database

The initial data obtained from the RNA sequencing were raw reads, with 64,336,475 raw reads obtained from the RNA sequencing transcripts of *B. gibsoni*. However, the raw data contained low-quality reads, adapter-related reads and reads containing N, which could not be used for further analysis. We therefore filtered the raw reads to remove these types of reads from the dataset and obtain clean reads. In this study, 62,580,653 clean reads were filtered from the raw reads, accounting for 97.28% of data, with an average data volume of 9.39 G. The average Q20 and Q30 values (percentage of bases with a quality score > 20 and > 30, respectively) and GC content were 96.79, 91.97 and 52.7%, respectively. The clean reads were then spliced to obtain the transcripts as references for subsequent analyses. However, as information of the *B. gibsoni* genome was not available at the time of the study, we had no reference genome for splicing and assembly. In this study, we used the “Trinity” tool for splicing the clean reads [37], and obtained 70,134 transcripts and 36,587 unigenes. The lengths of the transcripts and unigenes were analyzed, and most of the transcripts were found to be between 200 and 500 bp (57%); for the unigenes, we focused on those 500–1000 bp in length (29%). All of the unigene studies were performed with the gene functional annotation according to seven databases (NR, Nt [NCBI nucleotide sequences], Pfam [protein family], KOG, Swiss-Prot, KEGG and GO [Gene Ontology]). For GO classification, the unigenes were annotated in three major categories (Fig. 1): (i) biological processes; (ii) cellular components; and (iii) molecular functions. For the biological process category, most of the unigenes were matched to cellular process and metabolic process. For the cellular component category, most of the unigenes were assigned to cell and cell part. For the molecular function category, binding and catalytic activity accounted for the largest proportion. For KOG classification, the functions of “general function prediction only,” “posttranslational modification, protein turnover, chaperones” and “signal transduction mechanisms” were the dominant functions (Fig. 2). Most of the unigenes were predicted to be involved in signal transduction and translation processes in the KEGG classification. Through NR annotation, the unigenes could be matched to sequences that had a high similarity and had been deposited in the database to conduct gene functional annotation. Of the unigenes, 13.4% could be matched to *B. bigemina* genes and 2.8% could be matched to *B. bovis* genes. The clean reads were then mapped to the reference sequences that were the splicing results of Trinity by RESM software. The number of “readcount” of every gene obtained in this process was switched to FPKM. The FPKM value is currently the most common indicator for the expression level of unigenes [43–45]. The genes matched to the *Babesia* species were sorted based on the FPKM value (Additional file 3: Table S3). Of these, the gene with the highest FPKM value was the G-beta repeat domain-containing protein of *B. bigemina* (XP_012766105), followed by a hypothetical protein of *B. bigemina* (XP_012766347) and a hypothetical protein of *B. bovis* (XP_001611467). The amino acid sequences of these three proteins were all screened as query sequences and BLAST-searched in the uncomplete genome of *B. gibsoni*. For the query sequences of *B. bigemina* proteins (XP_012766105 and XP_012766347), there were no complete open reading frame (ORF) sequences when screening in the unassembled genome of *B. gibsoni*. After comprehensive analysis of the three sequences, we determined only to study the hypothetical protein of *B. bovis* (XP_001611467) in subsequent analyses.

### P30 of* B. gibsoni*

**Fig. 3:**
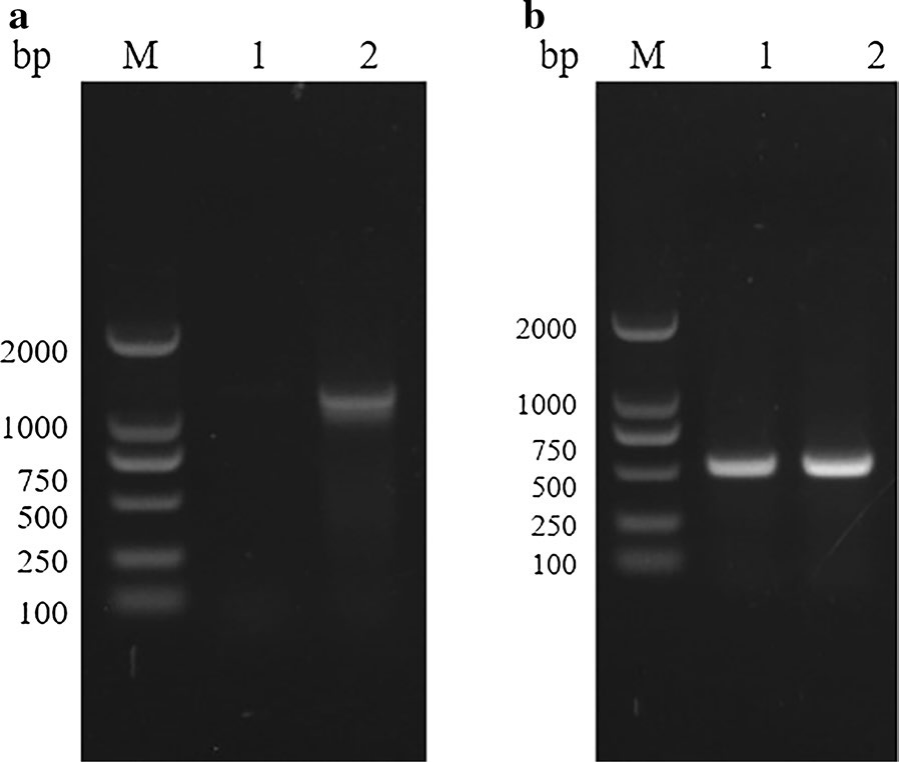
Amplification of the* B. gibsoni* P30 gene (Bg*P30*).** a** Lanes: M, marker; 1, cloned Bg*P30* gene from the complementary DNA (cDNA) of *B. gibsoni*; 2, cloned Bg*P30* gene from the genomic DNA (gDNA) of *B. gibsoni*.** b** Lanes: M, marker; 1, the truncated Bg*P30* gene cloned from gDNA; 2, the truncated Bg*P30* gene cloned from cDNA

**Fig. 4:**
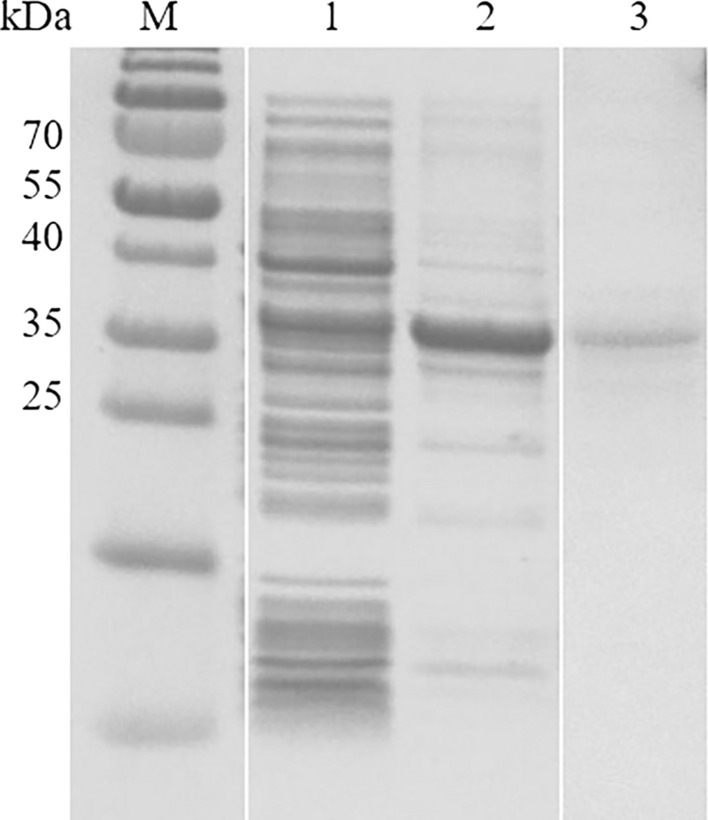
Sodium dodecyl sulfate-polyacrylamide gel electrophoresis analysis of protein expression of the recombinant BgP30 protein (rBgP30) in *Echerischia coli*. Lanes: M, molecular weight marker; 1, supernatant form of rBgP30; 2, inclusion body form of rBgP30; 3, purified form of rBgP30

The amino acid sequence of this hypothetical protein of *B. bovis* was screened in the genome of *B. gibsoni*. Through comprehensive analysis of the results of tBLASTn in the genome, the full-length of the 467-like gene in *B. gibsoni* was 1276 bp, comprising three exons, with lengths 120, 138 and 528 bp, respectively. Two introns, with lengths 31 and 459 bp, respectively, were interspersed among the three exons. The length of the ORF was 786 bp, encoding 261 amino acids with the predicted molecular weight of 30 kDa. The theoretical isoelectric point was 9.67. The 467-like gene in *B. gibsoni* was thus named *P30*. Forward and reverse primers were designed to clone the *P30* gene from the genomic DNA and cDNA of *B. gibsoni*, respectively. A specific band of approximately 1276 bp was amplified from the gDNA (Fig. 3). To further study this gene, the 528-bp exon was cloned from the cDNA and ligated to the pE-sumo vector. The pE-sumo-BgP30 was His-tagged with the predicted molecular weight of 32 kDa and was mainly expressed as the inclusion body at 37 °C (Fig. 4). The secondary structure of BgP30 was predicted and analyzed by online software. No potential signal peptides, transmembrane regions or GPI anchors were observed.

### Immunoreactivity of BgP30

**Fig. 5:**
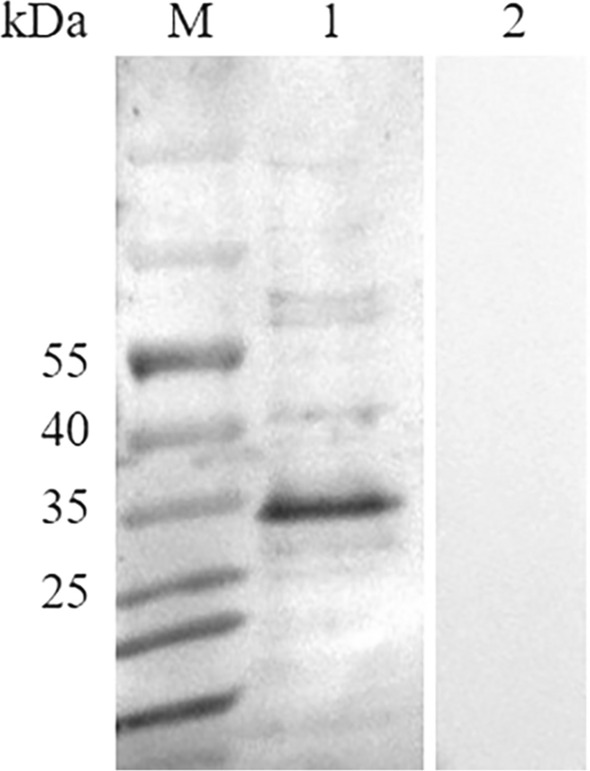
Determination of the immunogenicity of BgP30. Lanes: M, molecular weight marker; 1, rBgP30 incubated with serum of *B. gibsoni*-infected dog; 2, rBgP30 incubated with the serum of* B. gibsoni*-naïve dog

In the western blotting assay, rBgP30 was blotted onto the nitrocellulose membranes and then incubated with serum collected from healthy and *B. gibsoni*-infected beagle dogs, respectively. A corresponding band of about 32 kDa (consistent with the predicted molecular weight of rBgP30) was specifically recognized by the serum of the *B. gibsoni*-infected beagle dog, but not by the serum of the healthy beagle dog (Fig. 5). These results showed that P30 had good immunoreactivity that could effectively induce the production of antibodies in the beagle dog.

### Localization of P30 in* B. gibsoni*

**Fig. 6:**
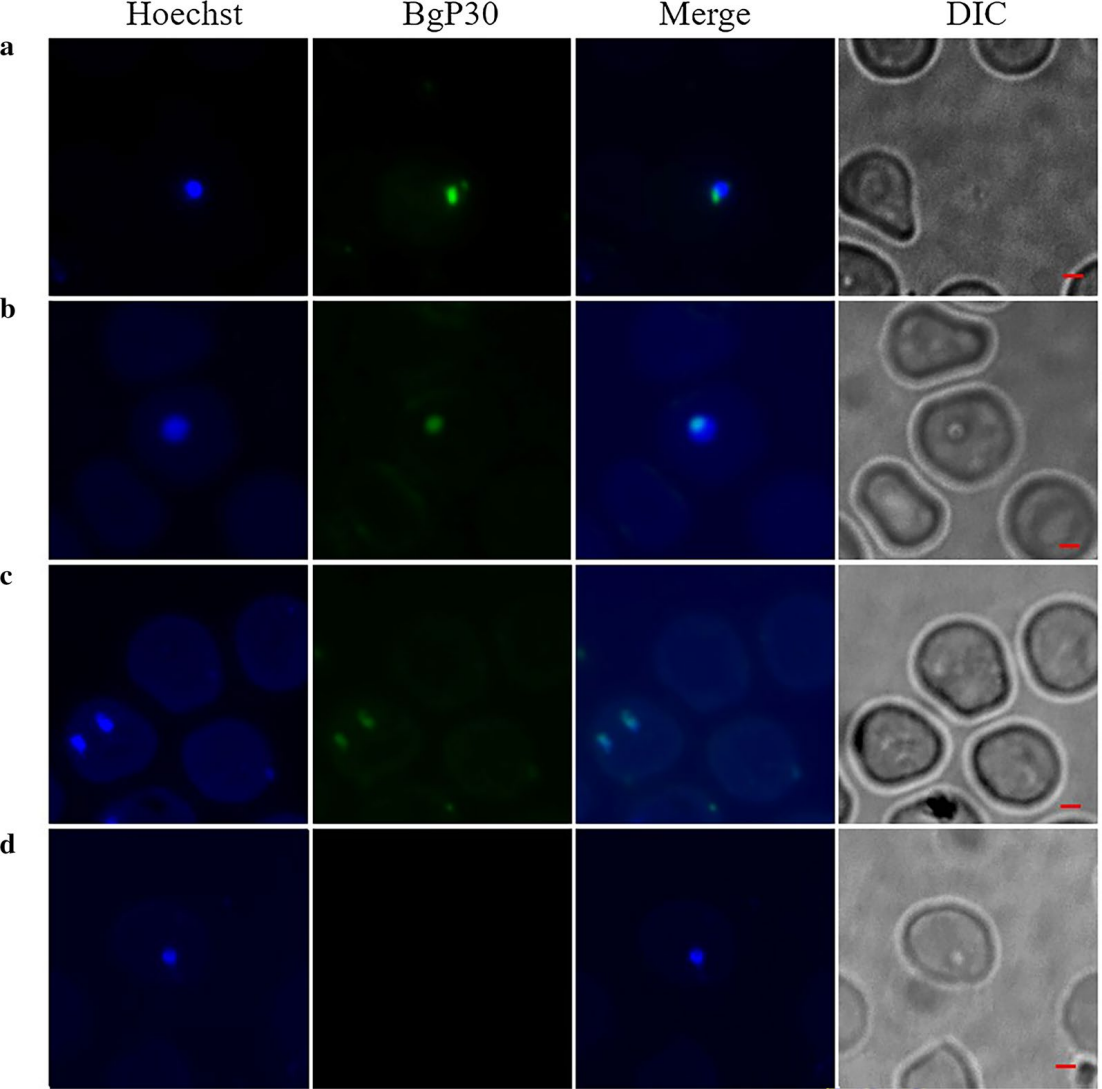
Cellular localization of the P30 protein in *B. gibsoni*. **a** During the extracellular stage, BgP30 with green fluorescence co-located with the nucleus of merozoites with blue fluorescence. **b** When the merozoite resided in the red blood cell, the poly-antibody against rBgP30 detected P30 in the intraerythrocytic merozoite of *B. gibsoni*. **c** At the post-invasion stage of paired parasites, P30 was also detected with green fluorescence adjacent to the merozoite nucleus. **d** Negative control: infected erythrocytes were incubated with the serum of * B. gibsoni*-naïve rabbits. Scale bar: 1 μm

The localization of P30 in *B. gibsoni* was determined by IFAT using the rabbit polyclonal antibodies against rP30. Both for extracellular and intracellular merozoites, the green fluorescence (P30 of *B. gibsoni*) simultaneously localized with the blue fluorescence (nucleus of *B. gibsoni*) (Fig. 6), but there was no green fluorescence detected by when rabbit polyclonal antibodies against rP30 were incubated with the serum of healthy rabbits.

### Determination of the antibody response of BgP30

**Fig. 7:**
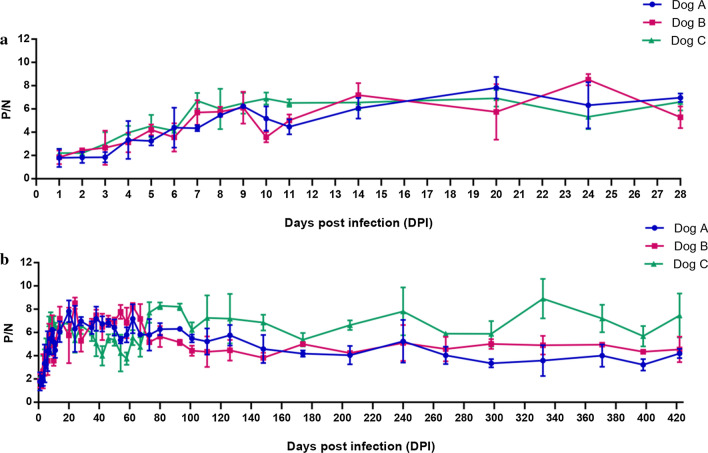
Detection of antibody response to rBgP30 in the serum of beagle dogs experimentally infected with *B. gibsoni,* by enzyme-linked immunosorbent assay. **a** Details of antibody response from day 1 to day 28 post infection. **b** Results for antibody response from day 1 to day 422 post infection. P/N, Mean OD of the test specimen reacted on antigen (P)/mean OD of the control serum reacted on antigen (N)

The serum of the experimental beagle dog that was infected with *B. gibsoni* was collected from day 1 to day 422 post infection to determine the antibody response of rBgP30 by ELISA. As shown in Fig. 7a, all three dogs were determined to have a detectable level of antibody response to rBgP30 on day 5 post infection and have a significant antibody response to rBgP30 on day 7 post infection. The antibody titer maintained a relatively stable value until day 422 post infection, even when the dog had reached the chronic infection stage with a low level of parasitemia (Fig. 7B).

## Discussion

*Babesia* spp. are included in phylum Apicomplexa, and compared to other members of this phylum, such as *Plasmodium*, which can invade both hepatocytes and RBCs, and *Toxoplasma gondii*, which can invade all nucleated cells, *Babesia* spp. can only invade RBCs, which have no nucleus, thereby providing space for hemoglobin, which accounts for up to 96% of the total protein in RBCs [46, 47]. Also, unlike *Plasmodium* and *T. gondii*, which will reside in a parasitophorous vacuole to separate themselves from the content of the host cell, research indicates that *Babesia* do not form a vacuole in the RBCs [46]. These differences suggest that *Babesia* have a simple life-cycle and a simple invasion mechanism that allows it to reside in the RBC. However, current knowledge on *Babesia* is relatively limited, and only the genomes of only a few *Babesia* species have been sequenced, including those of *B. bovis*, *B. divergens*, *B. bigemina*, *B. ovata*, *B. microti* and *Babesia* sp. Xinjiang [48–53]. For transcriptome studies, only the transcriptome of *B. bovis*, *B. divergens, B. ovata, B. microti* and* Babesia* sp. Xinjiang are available in databases [48, 49, 54, 55]. Comparison of these genomes suggests that the transcriptome will change to adapt with changes in the environment to a certain degree, in a manner that will better reflect gene expression. *Babesia* expresses different genes in response to its presence either in a vertebrate host or in ticks [56, 57]. Therefore, transcriptome data will greatly contribute to our understanding of the mechanism of how *Babesia* spp. can reside in RBCs. For *B. gibsoni,* one of the main agents of babesiosis in dogs, complete information on the genome and transcriptome is currently unavailable. Most of the current research on *B. gibsoni* has focused on antigen identification, drug treatment and the establishment of a related ELISA, PCR and/or other molecular level methods for diagnosis purposes. Some antigens have been identified as potential diagnostic candidates, such as P12 for serodiagnosis, and other *B. gibsoni* antigens, as P32, P38 and P50, which have been evaluated using ELISAs [31, 32, 36, 58, 59]. However, the mechanism for its asexual life in RBCs and sexual life in the vector tick remain unknown [60]. The unavailability of genome and transcriptome information has extensively increased research on diagnostic markers.

In this study, the transcriptome of *B. gibsoni* (Wuhan isolate) during the asexual stages in beagle dogs was sequenced and analyzed. A total of 62,580,653 clean reads and 36,587 unigenes were obtained. The unigenes obtained were then annotated by NR, Nt, KOG, Swiss-Prot, KEGG and GO analysis [61, 62]. The FPKM value, which is the common indicator for expression level, was used to screen some genes with relative high expression level in the transcriptome of *B. gibsoni* [43, 45]. The selected genes with high FPKM value as query sequences were successively BLAST-searched in the unassembled genome of *B. gibsoni*. A gene with a full length of 1276 bp was screened in the genome of *B. gibsoni* with the query sequence of high FPKM value of *B. bovis* (XP_001611467). The ORF of this gene was 786 bp and comprised three exons encoding 261 amnio acids. The predicted molecular weight of the encoded protein was 30 kDa, and the gen was named Bg*P30*. The amino acid sequence of the BgP30 protein was BLAST-searched in the sequences database in NCBI, and 13 sequences were identified with significant alignments, including proteins in *B. bovis* (XP_001611467), *B. ovata* (XP_028865573), *B*. sp. Xinjiang (XP_028872314), *Theileria equi* (XP_004832370), *Theileria orientalis* (UKK01334, XP_009691561, PVC49716 and UKJ88966), *Theileria parva* (XP_763348), *Theileria annulata* (XP_955014), *B. bigemina* (XP_012768809), *B. microti* (XP_012647546) and *Cryptosporidium andersoni* (OII78288), with *B. gibsoni* being the exception. All of the aligned sequences were hypothetical or uncharacterized proteins that have not been further studied and annotated with specific and detailed functions. The alignment results suggested that BgP30 had no homology with any sequences that had been annotated and studied in *B. gibsoni,* and that it was a new gene that could be further studied. It should be noted that the sequences with high similarities to BgP30 in other species had also not been deeply studied with regards to specific functions.

BgP30 was subjected to detailed assays in an attempt to elucidate related functions. In a first step, this protein was expressed as a His-tagged fusion protein in *E. coli*. The His-tagged protein was then used for antibody detection in subsequent assays. IFAT was conducted using antibodies against BgP30. The specific green fluorescence (indicator of BgP30) was detected in the *B. gibsoni* by IFAT, thereby confirming that P30 was present in *B. gibsoni*. These results confirm the western blotting assay can be used to specifically detect rBgP30 in the serum of beagle dogs infected with *B. gibsoni*, revealing that BgP30 could induce the production of antibodies in the host. Moreover, the serum *B. gibsoni*-infected Beagle dogs on day 5 post infection could even detect rBgP30, and the serum on day 7 post infection could significantly recognize the rBgP30 by ELISA. These results indicate that BgP30 has the potential to be an early diagnosis antigen for *B. gibsoni*; they are also consistent with the results of the transcriptome study showing a high expression level of BgP30 during the asexual stages in the RBCs of infected beagle dogs. Moreover, when the *B. gibsoni*-infected Beagle dogs were in the chronic stage of infection, BgP30 could be also recognized by the serum on day 422 post infection, suggesting P30 might be continuously expressed in *B. gibsoni* and may be considered as a good diagnostic candidate.

The growing significance of pets to humans and the ever increasing frequency at which dogs are a traveling companion to humans extensively increase the risk of *B. gibsoni* spreading. However, there is still no effective commercial diagnostic kit available for *B. gibsoni*. This study aimed to screen a candidate marker to be used for *B. gibsoni* diagnosis from the transcriptome of *B. gibsoni*. BgP30, with a high expression level, was confirmed by assays to be a potential candidate not only for early diagnosis but also during the chronic stage. The results of this study may be useful and provide the theoretical foundation for the development of diagnostic kits.

## Conclusions

To our knowledge, we report here the first results on the transcriptome of *B. gibsoni* endemic to Wuhan, China. Based on transcriptome results, BgP30 was screened for further study regarding its cellular localization and immunoreactivity. The results suggest that BgP30 can be regarded as a potential candidate for diagnostic purposes. These results may provide new insight into *B. gibsoni* research and promote the development of an effective diagnosis and control of *B. gibsoni* infection.

## Supplementary Information

**Additional file1: Table S1.** PCR primers for *Babesia* detection.

**Additional file2: Figure S2.** Parasitemia for each beagle dog.

**Additional file3: Table S3.** The list of genes that matched to the *Babesia* species.

## Data Availability

All data obtained in this study had been deposited to the National Center for Biotechnology Information (NCBI) with the accession number SRR12433308, SRR12433307 and SRR12433306. The GenBank accession number of P30 of *B. gibsoni* was OM925517.

## Abbreviations

cDNA: Complementary DNA
BLAST: Basic Local Alignment Search Tool
BSA: Bovine serum albumin
ELISA: Enzyme-linked immunosorbent assay
FPKM: Fragments per kilobase per million
HRP: Horseradish peroxidase
IFAT: Immunofluorescent antibody test
IPTG: Isopropyl-β-d-thiogalactopyranoside
LAMP: Loop-mediated isothermal amplification
PBS: Phosphate buffered saline
PPE: Percentage parasitized erythrocytes
rRNA: Ribosomal RNA
