# Supplementary figures and images for: A novel promising diagnostic candidate selected by screening the transcriptome of Babesia gibsoni (Wuhan isolate) asexual stages in infected beagles
Source: Parasit Vectors. 2022 Oct 10;15:362. doi: 10.1186/s13071-022-05468-4 (PMC9549657; doi:10.1186/s13071-022-05468-4)

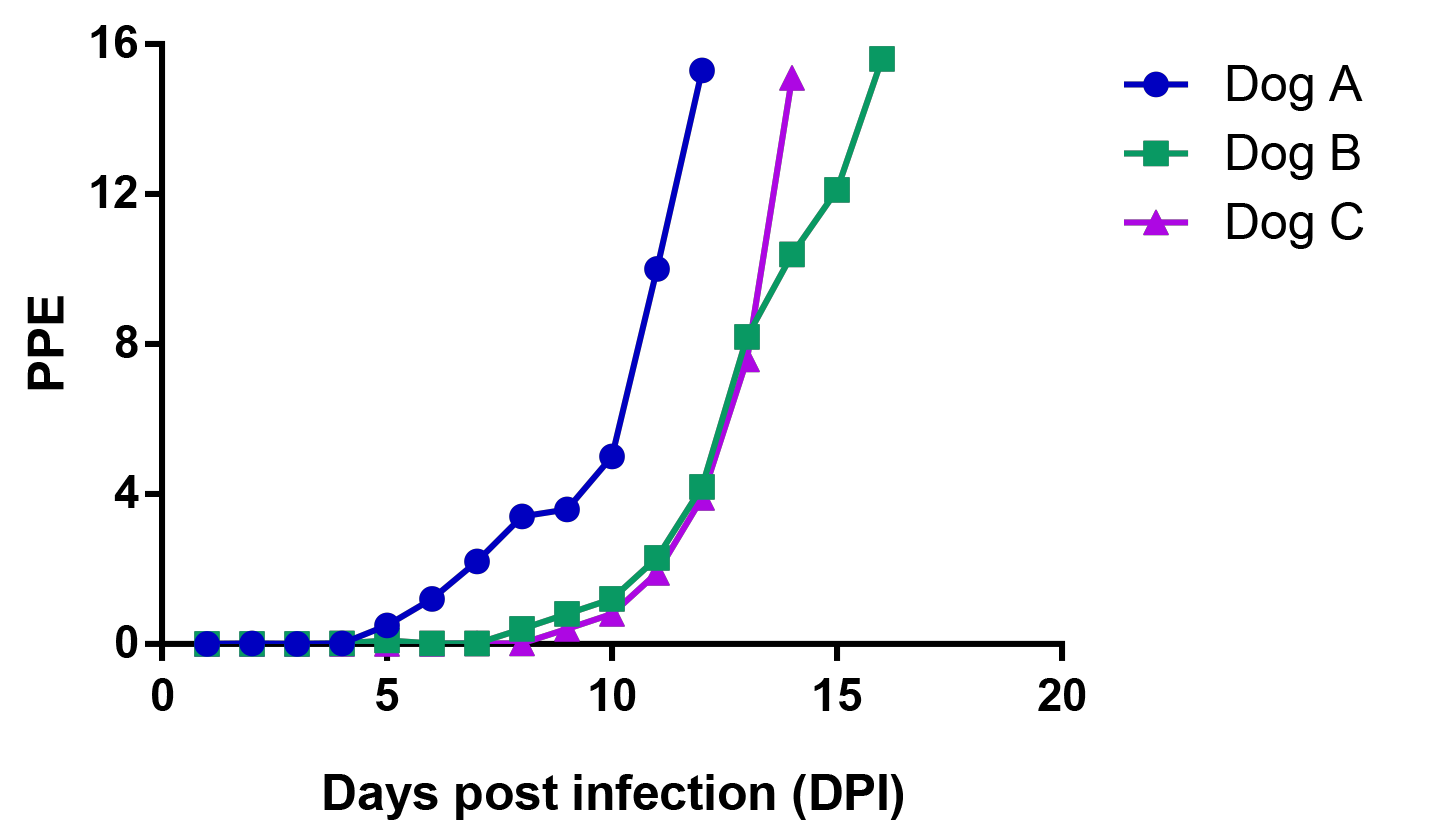

Supplement: Supplementary file 2 — Additional file2: Figure S2. Parasitemia for each beagle dog. [file 13071_2022_5468_MOESM2_ESM.tif]
